# Complex extensive urethral diverticulum on pelvic floor ultrasound and MRI

**DOI:** 10.1007/s00192-020-04355-9

**Published:** 2020-06-13

**Authors:** Victoria Asfour, Vik Khullar, Giuseppe Alessandro Digesu

**Affiliations:** 1 Kingston Hospital NHS Foundation Trust, Galsworthy Road, Kingston upon Thames, KT2 7QB UK; 2 St Mary’s hospital, Imperial College Healthcare NHS Trust, London, W2 1NY UK

**Keywords:** Urethral diverticulum, Stress incontinence, MRI, Pelvic floor ultrasound, Pain, Voiding dysfunction

## Introduction

A urethral diverticulum most commonly presents with recurrent urinary tract infection (51%), stress incontinence (45.5%), a vaginal lump (45%), urethral discharge (21%), and “the 3Ds” (dysuria, dyspareunia, post-void dribbling; 9%) [1]. Diverticula are on average 26 mm in diameter (range 8–45 mm) [1], and are U-shaped or circumferential in 84% [1].

## Case study

A 49-year-old patient presented with a 4-year history of severe urgency, frequency every 30 min, nocturia, but not incontinence. She described an almost continuous urgency (and sometimes pain) that did not get relieved with micturition. She experienced dyspareunia, dysuria and voiding dysfunction.

**Fig. 1:**
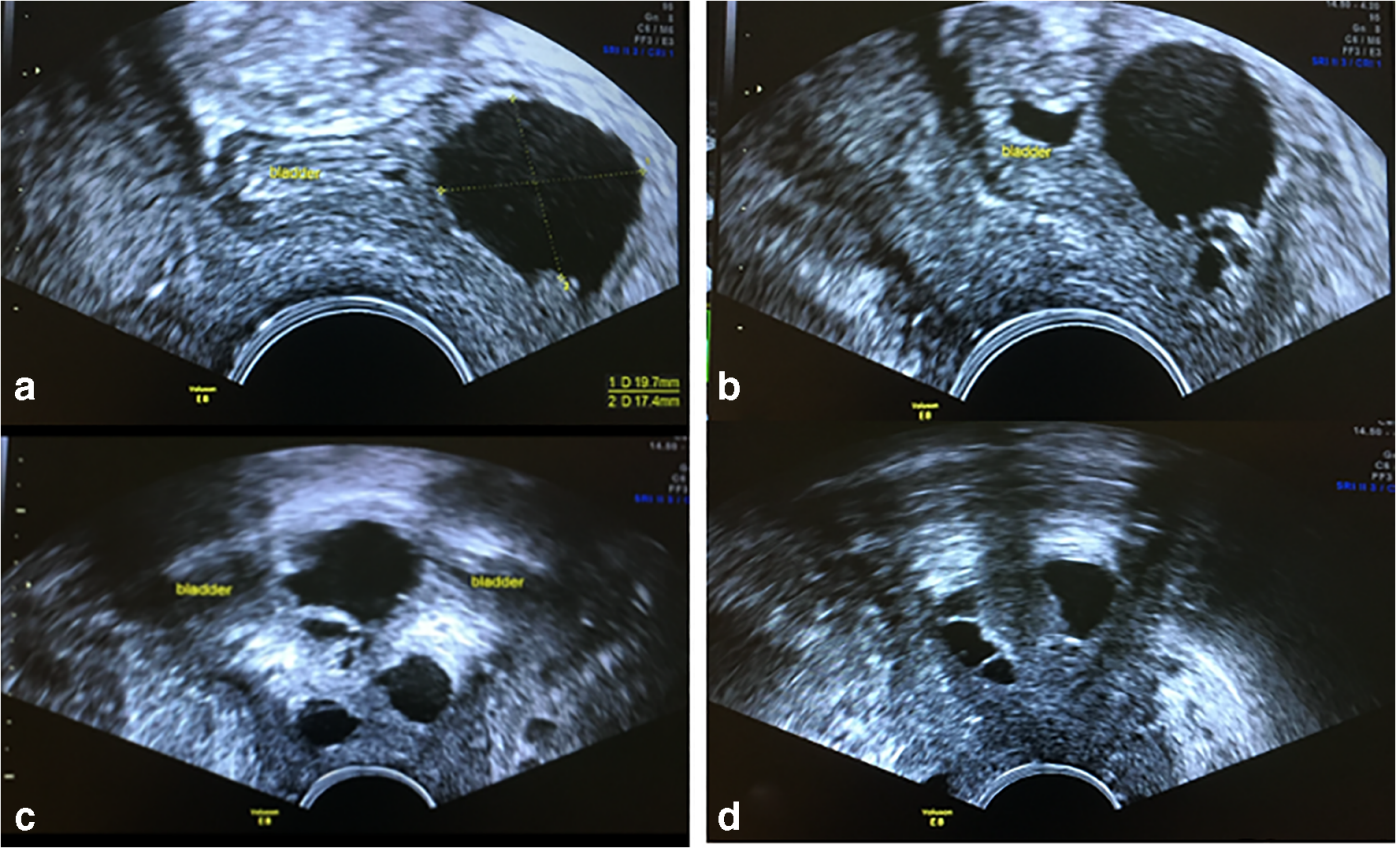
Transvaginal ultrasound showing cystic lesions (diverticulum) in the bladder wall and around the urethra. **a** Mid-sagittal image shows a cystic portion of the diverticulum in the bladder wall over the trigone. **b** Smaller cystic lesions inferior to the larger cystic structure are shown at the trigone. **c** Coronal images show multiple cystic lesions at the base of the bladder and along the proximal urethral length. The bladder is empty.** d** Coronal section showing cystic structures in the distal urethra

**Fig. 2:**
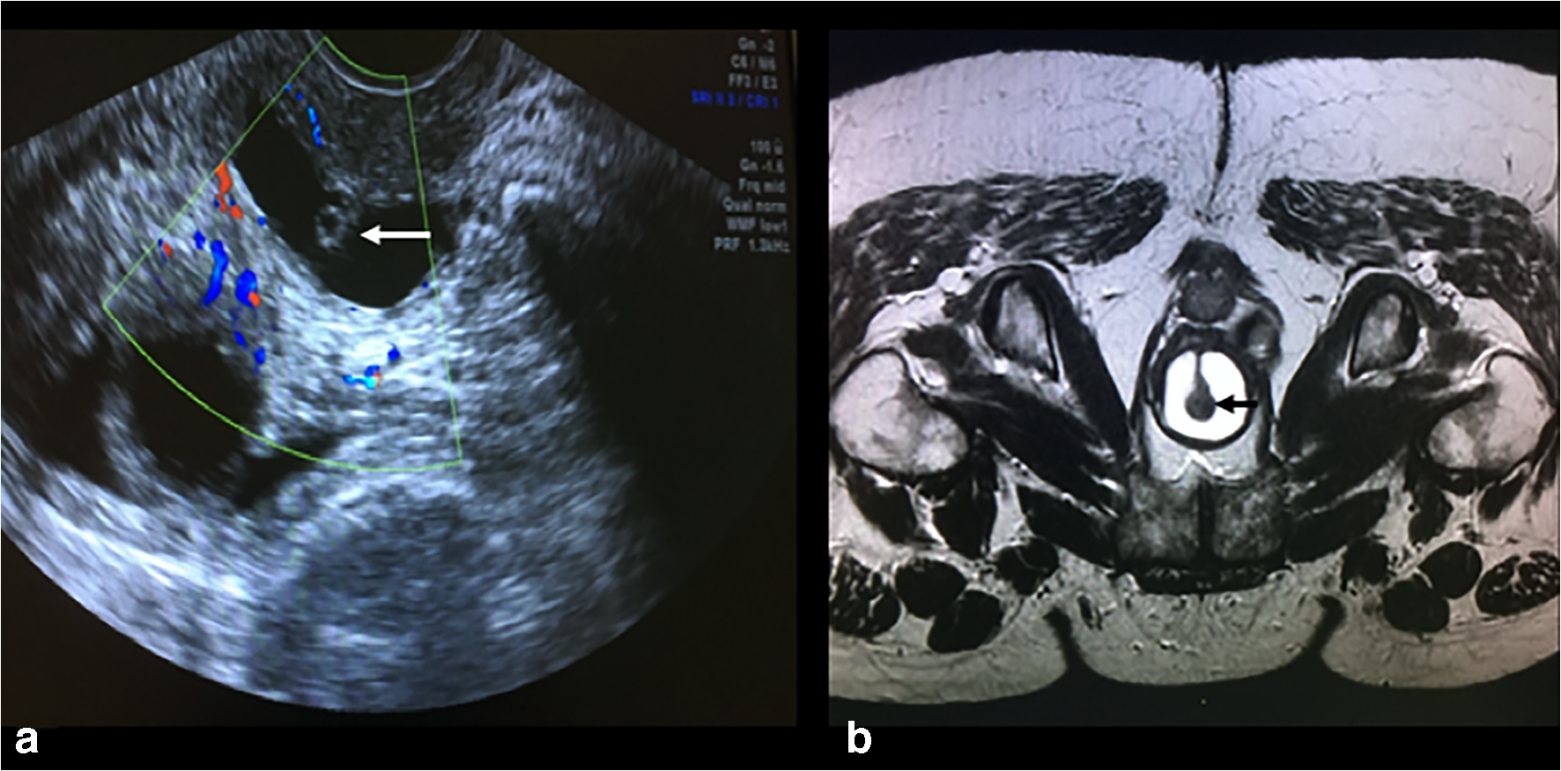
**a** Transvaginal ultrasound showing the urethra surrounded by the diverticulum. **b** MRI showing the same part of the urethra. The* arrows* demonstrate the urethra suspended within the diverticulum

**Fig. 3:**
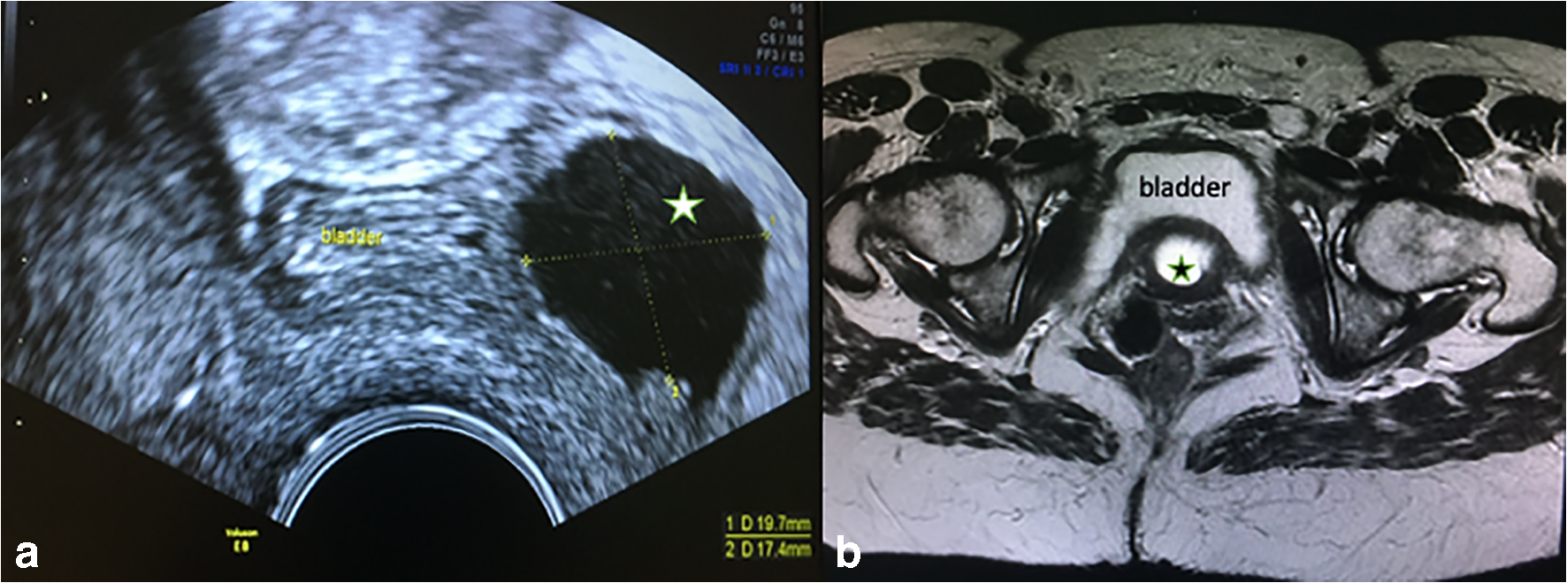
Magnetic resonance image at the level of the bladder above the urethra. The* star* demonstrates the fluid of the diverticulum

This was a tertiary level referral, as the patient had not experienced any improvement from assessment and management in other units for the last 2 years. At the time of referral, she had received first- and second-line medical treatments for overactive bladder and bladder installations. She had been previously investigated with urodynamics and cystoscopy. When she attended video urodynamics (VCU), she was tearful about the prospect of being catheterised, as this was always very painful. At this point, she was offered a pelvic floor ultrasound scan instead. The pelvic floor scan revealed an extensive cystic structure around the urethra (Fig. 1). MRI confirmed a urethral diverticulum in an extensive and unusual configuration, consistent with the pelvic floor ultrasound findings (Figs. 2, 3). Cystoscopy confirmed the opening to be at 7 o’clock at the mid-portion of the urethra.

The multidisciplinary urogynaecology team suggested resection of the urethral diverticulum with a Martius fat pad flap, to reduce the risk of post-operative stress incontinence. The patient opted for conservative management, because she was worried about the potential risk of stress incontinence post-operatively.

## Discussion

Stress incontinence may occur post-operatively, as urinary continence is a complex system that requires anatomical architecture of the urethral muscles, surrounding connective tissxues and neural feedback mechanisms [2]. A series of 100 diverticula managed with excision and a Martius flap, showed resolution of pre-existing stress incontinence in 59% and de novo stress incontinence in 14% [3].

## References

[1] Liu D, Qing Z, Wen L (2019). The use of tomographic ultrasound imaging on three-dimensional translabial ultrasound: a diagnostic sign for urethral diverticulum. Int Urogynecol J.

[2] Mistry MA, Klarskov N, DeLancey JO, Lose G (2020). A structured review on the female urethral anatomy and innervation with an emphasis on the role of the urethral longitudinal smooth muscle. Int Urogynecol J.

[3] Barratt R, Malde S, Pakzad M, Hamid R, Ockrim J, Greenwell T (2019). The incidence and outcomes of urodynamic stress urinary incontinence in female patients with urethral diverticulum. Neurourol Urodyn.

